# Clinic of Professor Dunglison

**Published:** 1845-09

**Authors:** Samuel G. White

**Affiliations:** Georgia


### CLINICAL LECTURES AND REPORTS.

### PHILADELPHIA HOSPITAL.

*Saturday, February* 15, 1845.

### CLINIC OF PROFESSOR DUNGLISON.

Reported by Dr. Samuel G. White, of Georgia.

In the commencement of the lecture, the attention of the class was directed to the progress of the cases which had previously been under consideration. The first of these was that of the patient labouring under CHOREA. He has very much improved; and although slight movements of the affected limbs remain, he may be discharged, provided the treatment be continued. It is very important, in all cases of a chronic nature, that the use of the remedies should be prolonged for several weeks, in order to effect permanent good.

The patient who suffered from OBSTRUCTION OF THE BOWELS, which was supposed to be owing to the existence of stricture about the sigmoid flexure, has also greatly improved. The treatment has consisted almost entirely in the administration of enemata of soap suds. On Saturday last, nearly half a gallon of this fluid was thrown into the colon by means of a stomach tube. This was followed by the evacuation of numerous large pellets of indurated fæcal matter, which were probably the cause of the obstruction. Since this, the individual has continued to improve, and has experienced no return of the painful spasms of the intestines, under which he before suffered. He will now, doubtless, recover, although this could scarcely have been anticipated a few days ago. It will be important to prevent the re-accumulation of fæces, which may be effected by continuing the daily use of the enemata. As he is somewhat anæmic, it will be well to give him a chalybeate, as 20 grains of the ferri sub-carbonas, three times daily. Should this produce, or be accompanied by constipation, a few grains of rhubarb, or some other laxative, may be combined with it. This case is very instructive, as exhibiting the importance of carefully investigating the true nature of disease, and of adapting the appropriate remedies. It was one of constipation, but so obstinate that it would have resisted all ordinary cathartic agents, and was successfully treated only by a thorough appreciation of its cause. The disease—as the class have observed—was treated altogether *per anum.*

The lecturer took occasion to recommend the class to provide themselves with a proper apparatus for throwing fluid into the colon. In country practice, this is often a great desideratum. By the common glyster pipe, the injection can rarely be made to pass higher than the rectum, and therefore can have no influence on hardened masses of fæces situate in the cæcum and colon. But this difficulty is readily overcome by introducing a large rectal tube beyond the sigmoid flexure, and injecting the fluid by a properly adapted syringe. In this manner, the whole colon may be readily distended. In his own person, the lecturer remarked, he could readily trace the course of the colon by percussion, after it had been filled by an injection.

The Professor next made a few observations on a case of

CHRONIC GASTRITIS.

The patient—a male—has been for a length of time affected with PYROSIS, but within a few days he was attacked with vomiting, and an increase of all his symptoms. The matter ejected consisted chiefly of a watery mucous fluid. It was very ropy, and the result, no doubt, of irritation, if not of positive inflammation of the mucous follicles.

Pyrosis, under which he so long laboured, is merely a form of indigestion, characterized by the evacuation or regurgitation of quantities of watery, more or less slimy fluid, possessed of different sensible characters—being sometimes bland, and at others acrid or irritating. It is not an unfrequent attendant on the gastric disease.

Although, in this case, there has been a marked increase in the general symptoms, there is very little in those of gastritis. This will admit of easy explanation. The mucous membrane of the stomach, like all others, although highly organized, is not possessed of a very exalted sensibility to mechanical agents. This was noticed in the case of Dr. Beaumont's servant—San Martin—whom the lecturer had frequent opportunities of seeing. In that case, considerable pressure could be exerted on the interior of the stomach by means of a bougie, introduced through the opening in the abdominal parietes, without exciting any positive pain,—a disagreeable sensation of pressure being alone experienced. This insensibility of the gastric mucous membrane is further evinced by the impunity with which the most heterogeneous and often irritating substances are taken into that viscus. Hence it is, that even when inflamed, the sensibility of the organ may not be highly exalted. Not unfrequently, indeed, decided inflammation of the gastro-enteric surface exists, without the development of any very marked local phenomena.

In the case under consideration, however, there is sufficient pain experienced, when pressure is made over the abdomen, to indicate the existence of irritation, if not of inflammation. In conjunction with this, the tongue is red and slightly furred, showing a morbid condition of the membrane.

The lecturer here cautioned the class against placing too much reliance on the red color of the tongue as an unfailing sign of gastritis, as it is—in his experience—as frequently white.

From the chronic character of the gastric affection in this case, it might be questioned whether it were not connected with some *vice*, as carcinoma. The general appearance, and other phenomena, however, render this improbable.

The treatment, in this case, will consist in making counter-irritation over the epigastrium with the unguentum antimonii, and the internal use of charcoal with magnesia. This combination is often beneficial—the charcoal acting as a tonic, whilst the magnesia neutralizes redundant acid, and by its laxative proper-

50*

ties removes any irritating matters that may exist in the canal. Its effect will be reported on a future occasion.

In connection with gastritis, the professor made a few remarks on

### GASTRORRHŒA.

This term is applied to a profuse discharge of mucus or watery fluid from the stomach, and by some is considered to be always inflammatory in its nature. This view of its pathology, however, is not borne out, inasmuch as, in the majority of instances, there are no evidences of the presence of inflammation, unless the increased secretion be so regarded. It can easily be comprehended, that by any irritation of the gastric surfaces its secretion may be increased, and it is not uncommon to meet with cases of very profuse secretion from the mucous membranes, without the least sign of inflammatory excitement. There seems to be a simple *gleet* of the gastric mucous surface. Sometimes, however, gastrorrhœa may unquestionably be dependent on inflammation, and then it must be treated accordingly. Where there is no excitement, the treatment will consist principally in the use of astringents. It should be remembered, that inflammation of the mucous membrane is pathologically the same wherever it may be seated, and demands for its relief the same therapeutical agents. Taking this view of the disease, our ideas of different mucous inflammations will be simplified, and the proper treatment be better understood.

The professor next introduced a female labouring under an affection very analogous to that just alluded to—

### BRONCHORRHŒA.

This is characterized by a profuse discharge from the bronchia, resembling much the fluid of gastrorrhœa. Bronchorrhœa has been considered, but erroneously, by some to be identical with chronic bronchitis. The fluid, however, is different; that discharged in bronchitis being a vitiated mucus, whilst this is watery and thin, with but little mucous admixture.

[The expectoration of the patient was here exhibited to the class. It was considerable in quantity, and served well to illustrate the nature of the secretion in this affection.]

Bronchorrhœa is not of such common occurrence as gastrorrhœa, or chronic bronchitis, and may be regarded as a rare disease. The treatment might consist in the employment of such means as are proper in gastrorrhœa. It very frequently happens, however, that the morbid condition of the bronchial membrane is connected with some more serious organic disease of the lungs or heart; and the same benefit, consequently, could not be expected to result from the treatment.

In the case under consideration, from the diminished percussive resonance and respiratory sound under the right clavicle, and the exaggerated murmur under the left, it is very probable that consolidation of the pulmonary tissue exists at the apex of the right lung. But it is not easy to determine whether this preceded, or has any connection whatever with the bronchorrhœa. It is probable, however, that the disease of the mucous membrane is independent of the condition of the lungs. The treatment will, therefore, consist in establishing counter-irritation over the chest, with the inhalation of some excitant vapour. By this means our remedies are made to come into immediate contact with the diseased surface, and are thus able to exert a more potent influence over it. With this view, the vapour of iodine, associated with that of conium to render it less irritating, will be prescribed. Should this fail, however, chlorine, which may be disengaged from one of its combinations, as chlorinated lime or chlorinated soda, will be substituted for iodine. The effect of these applications is often exceedingly beneficial, and not unfrequently succeeds in removing the disease when all other means have proved unsuccessful. In this case, the constitution of the patient has not suffered to any extent, and the disease will probably terminate favourably. At times, however, hectic is developed, in consequence of the profuse secretion and constitutional irritation; and if complicated with tuberculosis, or other serious organic lesion, it may prove fatal.

The lecturer then proceeded to present some observations on

### RHEUMATISM.

He remarked, that at present there seemed to be a peculiar condition of the atmosphere—a sort of *constitutio aeris*—to use the language of Sydenham—which is inappreciable—favourable to rheumatic affections. There appears, too, to be a tendency to certain articulations rather than to others, as in nearly every case the shoulder joint is affected. He reminded the class that he considered rheumatism to be largely neuropathic, and certainly not identical with ordinary acute inflammation.

In the case reported some weeks since, a perfect cure was effected by means of sulphate of quinia. This agent has proved no less successful in numerous other cases that have since occurred in the hospital. In the vast majority, it was sufficient of itself to effect the cure; but in a few, from the unusual degree of vascular excitement, the application of cups to the spine was advisable. This situation for topical bleeding was selected, not from a supposition that the medulla spinalis is diseased in such cases, but from its convenience, and its being a most sensitive surface. Sometimes it becomes necessary to give large doses of opium to allay the pain and excitement. This agent, being a powerful narcotic and sedative, exerts its influence—the professor thinks—much in the same manner as quinia, which, in large doses, is markedly narcotic and sedative. It will be found exceedingly beneficial in many cases of acute rheumatism, and is highly recommended, as a most powerful remedy in the disease, by Dr. Christison. The professor here desired the class not to be misled by the opinion that opium is always a stimulant. This belief has been long held by many, but is unquestionably erroneous. In small doses it is certainly a stimulant, but in large ones its sedative action is manifested in a marked manner. Where sedation is desired, it should never be given in less than 2½ or 3 grain doses. Aconite acts in a similar manner, and has been highly extolled by many.

The patient, a female, who was now introduced before the class, had been labouring under rheumatism of the shoulder, but not of a very acute character. She was put on the use of sulphate of quinia on entering the house, and under it alone has experienced marked relief. The lecturer thinks that it may be given fearlessly in every case. He has never witnessed any injurious effects from its employment, and, as before observed, he regards it in full doses a potent sedative. It has been prescribed in every stage of intermittent, without producing any signs of a stimulating agency. Even the pulverized cinchona has been employed, without any injurious effects, in the hot stage of that fever; although, from its containing much indigestible matter, as woody fibre, it might have been inferred that it would prove exciting, and consequently deleterious.

Before administering the sulphate of quinia in these cases, it may be well to pave the way for its action by some mild cathartic, although this is not indispensable. The dose of the sulphate, which may be 18 or 20 grains daily, may be gradually increased, care being taken to suspend it on the supervention of any symptoms indicative of its influence on the nervous system. When the excitement in acute rheumatism is very high, it will be proper to resort to depletion; but this should not be carried too far. The plan of treating rheumatic affections by the sulphate of quinia the professor has found extremely successful, both in private and public practice.

### PATHOLOGICAL SPECIMENS.

In conclusion, the professor directed the attention of the class to several very interesting morbid specimens. The first of these was obtained from a female, who died shortly after her entrance from CANCER OF THE UTERUS.

From the lemon yellow colour of her countenance, connected with the great fœtor of her person, and a discharge from the vagina, the lecturer had no hesitation in pronouncing this, at first, to be a case of cancer.

She was, when he first saw her, labouring under complete suppression of the renal secretion, and had passed no urine whatever for twelve days. There was no evidence, at least, that any was secreted during that time.

In most cases of this character, the principles of the urine are separated by some other part of the system, as by the skin, mucous membranes, &c. Sometimes the secretion takes place from the meninges of the brain, and very serious or even fatal effects are the consequence of the presence of the urinous fluid in the encephalon. It is remarkable, that this important depuratory process should have been suspended so long in this case without more serious results. The patient recovered from the suppression, but died shortly afterwards, worn out by the cancerous affection.

On making the necroscopy, one of the kidneys was found considerably enlarged, and modified in character, so that the cortical was not easily distinguishable from the tubular portion. In the cavity of the uterus several scirrhous tumors were found near its fundus, and an open cancer at its lower part, which had destroyed the cervix and os uteri, and extended to the upper portion of the vagina.

[The morbid specimen was here passed to the class for inspection, as it exhibited well one form of cancer.]

Cancer belongs to the class of cachexiæ, or affections dependent on some *vice* in the system. Generally, when fully formed, it is impossible to remove this vice by any treatment whatever.

All cancerous tumors have been referred to one of three varieties—colloid, encephaloid, or scirrhus. Of these, the encephaloid is the most inveterate, and although it may be removed by an operation, it is exceedingly liable to return in some other part, and terminate fatally.

It is a curious fact, that cancerous growths are developed by cells, which receive their nourishment from the surrounding tissues, and possess the power of self-generation. If one of these cells be introduced into a second individual by inoculation, the disease may be, like small pox, communicated. From the power of reproduction possessed by these cells, the system of the individual becomes completely impregnated with the diseased matter, and cannot be freed from it. For this reason—as well as from the results of extensive statistical inquiries into the results of operations for cancer—the propriety of removing these growths has been recently much questioned; and it is now agreed, by the best authorities, that it is better not to interfere with them. If the local affection be not removed by the surgeon, the disease may continue for a great length of time, and the individual enjoy life, without much suffering, for several years. The lecturer referred to a case which he had recently attended, of a female upwards of 80 years of age, who had suffered for, he believes, more than 25 years with open cancer of the mamma.

When an operation is practised at all, it ought to be at the earliest possible period, and before the constitution is saturated, as it were, with the cancerous cachexy.

The professor, regards cancerous affections as a diseased condition of the system of nutrition, and as such, to be removed, if at all, by agents that modify that process. As in all other cachexiæ, recourse must be had to articles belonging to the class of eutrophics, or remedies which, by being received into the circulation, may change the condition of the blood, and, through it, modify the action of the tissues which it bathes. Unfortunately, however, no permanent good can be expected from any treatment. We may prolong life, and alleviate the sufferings of the patient, but cannot eradicate the disease.

Cancerous affections of the uterus are by no means unfrequent, and, as before stated, generally terminate fatally. It is our duty, however, to endeavor to do all that is practicable to render the patient comfortable to herself and to others. Hence, for correcting the fœtor, which is always great, injections of various disinfectant agents should be employed. Of these, the chlorinated preparations, and the acetate or simple sulphate of alumina, are to be preferred.

The other viscera in this case, with the exception of the liver, which was much lighter colored than usual—the effect of hard drinking—were in a healthy state.

Professor Dunglison concluded by exhibiting to the class a marked specimen of TUBERCULOSIS OF THE LUNG, on which he did not dwell, as he had presented already so many specimens of a similar kind before the class.

